# 922. The Impact of Clinically Significant CMV Infections on Other Viral Infections in the Era of Letermovir Primary Prophylaxis

**DOI:** 10.1093/ofid/ofab466.1117

**Published:** 2021-12-04

**Authors:** Amy Spallone, Krithika Srinivasan, Joseph Sassine, Terri Lynn Shigle, Victoria Handy, Jeremy Ramdial, Fareed Khawaja, Ella Ariza Heredia, Roy F Chemaly

**Affiliations:** 1 Baylor College of Medicine, Houston, Texas; 2 University of Texas MD Anderson Cancer Center, Houston, Texas; 3 The University of Texas MD Anderson Cancer Center, Houston, TX

## Abstract

**Background:**

Cytomegalovirus (CMV) is a frequent complication after hematopoietic cell transplant (HCT) and may increase the risk of other viral infections through its immunomodulatory effects. Letermovir, a novel antiviral targeting the viral terminase complex, was approved for primary prophylaxis in CMV-seropositive adult recipients after allogeneic HCT (allo-HCT). Because of its efficacy and safety, letermovir has become the standard of care for primary prophylaxis against CMV during the first 100 days post-transplant. However, its impact on the frequency of other viral infections and non-relapse mortality (NRM), through its reduction in clinically significant CMV infections (CS-CMVi), is not known.

**Methods:**

This is a single-center, retrospective cohort study of 150 allo-HCT recipients, including controls that were matched by the transplant type (match-unrelated, matched-related, cord, and haploidentical), cared for at our institution between March 2016 and December 2018. Baseline demographics, transplant characteristics, prophylaxis, CMV and other viral infections, and outcomes were collected (Table 1) and analyzed on IBM® SPSS version 26 using a binary logistic regression model for multivariate analysis. For univariate analysis, we used Chi-square and Fischer’s Exact Test.

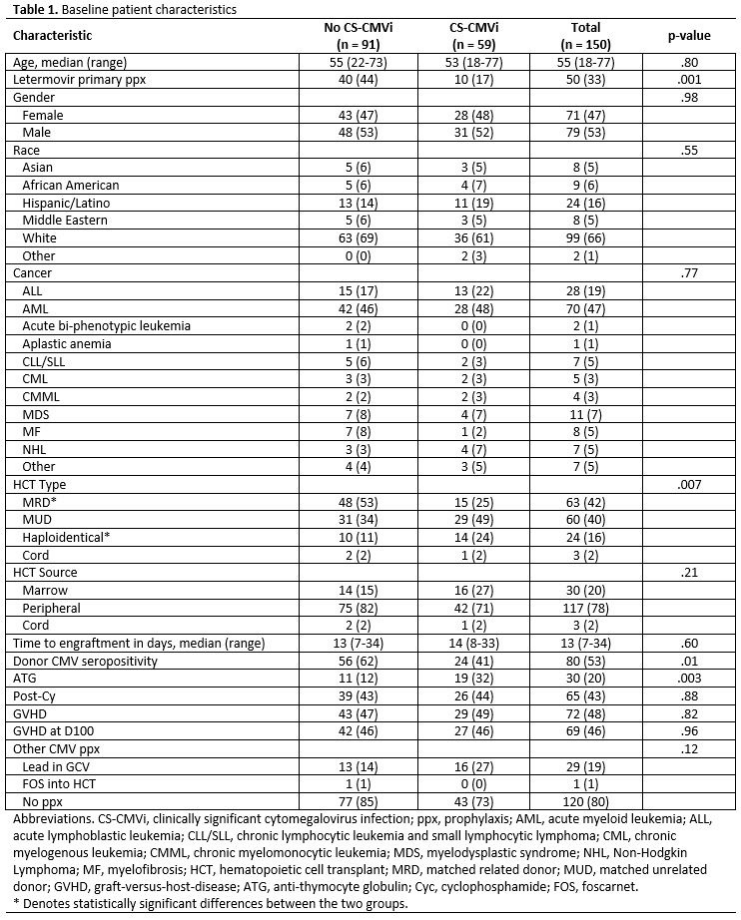

**Results:**

In our 2:1 matched cohort analysis, 50 patients received letermovir for primary prophylaxis during the first 100 days post-HCT, and 100 did not. In a univariate analysis with CS-CMVi as the outcome, there was a statistically significant difference in NRM at 24 and 48 weeks. Our data indicated a trend towards a decrease in other viral infections for those without CS-CMVi (Table 2). However, in a multivariate analysis accounting for primary prophylaxis with letermovir as an effect modulator, CS-CMVi did not demonstrate a significant impact on the frequency of other viral infections but was associated with NRM at week 24 and 48 (Table 3). Interestingly, having ALL and donor CMV seropositivity were protective factors against other viral infections (*Herpesviridae*).

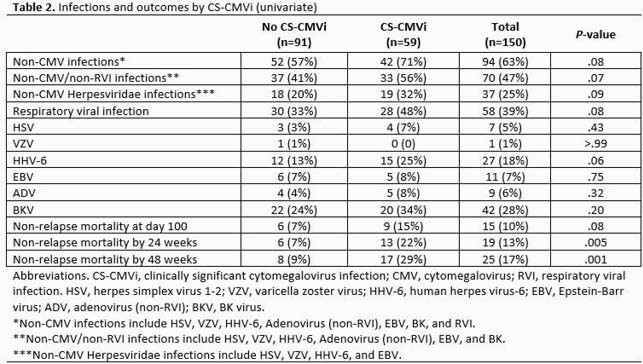

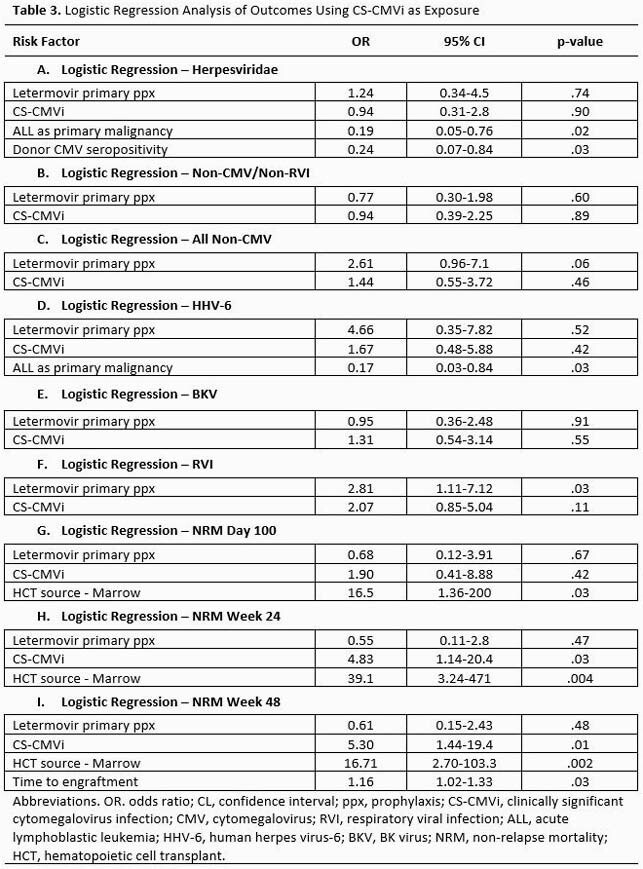

**Conclusion:**

Our study showed that CS-CMVi is associated with higher 24- and 48-week non-relapse mortality but with no increase in the incidence of other non-respiratory viral infections in this matched cohort of allo-HCT recipients.

**Disclosures:**

**Fareed Khawaja, MBBS**, **Eurofins Viracor** (Research Grant or Support) **Ella Ariza Heredia, MD**, **Merck** (Grant/Research Support) **Roy F. Chemaly, MD, MPH, FACP, FIDSA**, **AiCuris** (Grant/Research Support)**Ansun Biopharma** (Consultant, Grant/Research Support)**Chimerix** (Consultant, Grant/Research Support)**Clinigen** (Consultant)**Genentech** (Consultant, Grant/Research Support)**Janssen** (Consultant, Grant/Research Support)**Karius** (Grant/Research Support)**Merck** (Consultant, Grant/Research Support)**Molecular Partners** (Consultant, Advisor or Review Panel member)**Novartis** (Grant/Research Support)**Oxford Immunotec** (Consultant, Grant/Research Support)**Partner Therapeutics** (Consultant)**Pulmotec** (Consultant, Grant/Research Support)**Shire/Takeda** (Consultant, Grant/Research Support)**Viracor** (Grant/Research Support)**Xenex** (Grant/Research Support)

